# Synthesis and Antimicrobial Evaluation of Some Heterocyclic Chalcone Derivatives

**DOI:** 10.3390/molecules16032304

**Published:** 2011-03-09

**Authors:** Nagwa Mohamed Mahrous Hamada, Essam Mohamed Sharshira

**Affiliations:** 1 Department of Chemistry, Faculty of Education, Alexandria University, Alexandria, Egypt; E-Mail: nagwahamada2002@yahoo.com (N.M.M.H.); 2 Department of Chemistry, Faculty of Science, Alexandria University, Alexandria, Egypt

**Keywords:** chalcones, isoxazoles, pyrazoles, oxadiazoles, antimicrobial

## Abstract

Some new heterocyclic compounds containing isoxazole, pyrazole and oxadiazole ring systems were prepared from various chalcones. The synthesized compounds have been characterized by elemental analysis and spectral methods. These compounds were screened for their antimicrobial activities.

## 1. Introduction

Chalcones are synthesized by condensing ketones with aromatic aldehydes in the presence of suitable bases. They are very useful intermediates for the synthesis of five- [1,2], six- [1,3] and seven-membered [4] heterocyclic compounds. Chalcone derivatives exhibit diverse pharmacological activities [5,6,7,8,9,10,11,12,13,14]. It is therefore, not surprising that many synthetic methods have been developed for the preparation of heterocycles starting from chalcone precursors that have been tested for their antimicrobial activities.

## 2. Results and Discussion

**Scheme 1:**
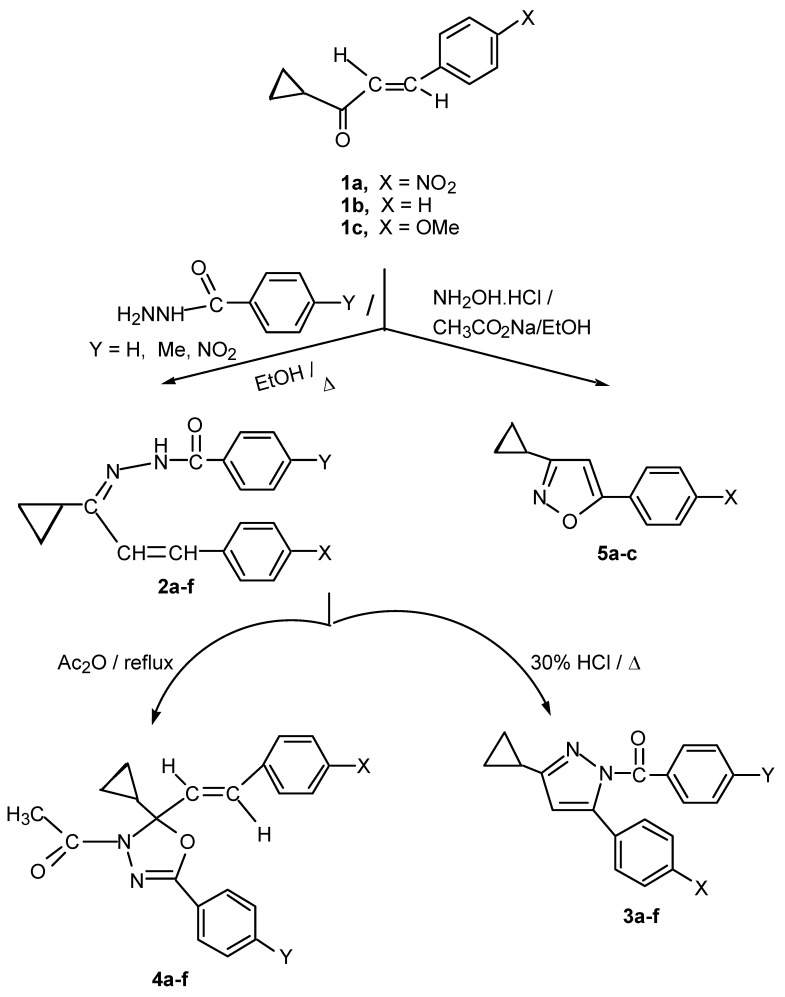
Synthesis of **2a–f**, **3a–f**, **4a–f** and **5a–f**.

All of our results are shown in **Scheme 1**. The starting chalcones **1a–c** were obtained in good yields by a base catalyzed condensation [15-16] of appropriately substituted benzaldehydes and cyclopropylmethyl ketone [17]. The method is attractive since it specifically generates the (*E*) isomer [18].

The hydrazones **2a–f** were prepared by the reaction of chalcones **1a–c** with benzoyl hydrazine derivatives and were subsequently used for the syntheses of various pyrazoles **3a–f** and oxadiazoles **4a–f**. The IR spectra of these hydrazones revealed the characteristic bands for vinyl CH=CH at 1582–1617, C=N at 1616–1647, C=O at 1664–1698 and NH at 3330–3420 cm^−1^. The ^1^H-NMR spectra showed the presence of a singlet at δ = 9.97–10.82 ppm for the NH proton, a multiplet at δ = 7.17–8.45 ppm characteristic for the aromatic protons and the olefinic =C–C*H*=CH, a doublet at δ = 6.60–6.95 ppm characteristic for the olefinic =C–CH=C*H* proton. The cyclopropyl ring protons appeared as two multiplets in the range δ = 1.56–2.64 ppm (CH) and δ = 0.70–1.46 ppm (2CH_2_), respectively.

The pyrazole derivatives **3a–f** were obtained by treatment of hydrazones **2a–f** with 30% hydrochloric acid. The IR of **3a–f** showed the characteristic bands for C=C–Ar at 1519–1596, C=N at 1623–1644 and amide carbonyl band at 1660–1686 cm^−1^, while the ^1^H-NMR spectra showed a singlet at δ = 6.64–7.12 ppm for the pyrazole–C_4_–H. On the other hand, refluxing of hydrazones **2a–f** with acetic anhydride gave the corresponding dihydro-1,3,4-oxadiazole derivatives **4a–f**. The mechanism of cyclization reaction has been well studied [19-20]. The IR spectra of the dihydro-oxadiazoles **4a–f** lacked the NH, but showed a carbonyl absorption at 1667–1677 cm^−1^ for the acetyl group. Their structures were further confirmed from the ^1^H-NMR spectra which does not reveal the presence of NH signal present in the starting hydrazone **2**, moreover, the ^1^H-NMR of **4** exhibited a singlet of three protons intensity at δ = 2.10–2.16 ppm for the COCH_3_. Finally, treatment of chalcones **1a–c** with hydroxylamine hydrochloride in presence of sodium acetate produced isoxazoles **5a–c** in moderate yield. The structure of **5** was fully confirmed by spectral method. For example, the IR of **5** does not show the presence of carbonyl band characteristic for the starting chalcone **1**. The ^1^H-NMR of **5** exhibited a singlet of one proton intensity at δ = 5.42–5.69 ppm characteristic for the isoxazole–C_4_–H. Melting points, elemental analysis and spectral methods are outlined in **Table 1** and **Table 2**.

### 2.1. Antimicrobial Activity

All the synthesized heterocyclic derivatives, pyrazoles **3a–f** oxadiazoles **4a–f** and isoxazoles 5a–c were assayed for their antimicrobial activity against four test organisms: *Staphylococcus aureus* ATCC6538P, *Escherichia coli* ATCC8739, *Pseudomonas aeruginosa* ATCC9027 and *Candida albicans* ATCC2091 using rifampicin (5 μg/disc) and ampicillin (10 μg/disc) as standard drugs following agar well-diffusion method [21].

The tested heterocyclic compounds showed no significant effect against *Pseudomonas aeruginosa* and *Candida albicans,* whereas they showed a potent activity against *Staphylococcus aureus* and *Escherichia coli.* The maximum activity (+ + +) (MIC = 25 μg/mL) was indicated for compounds **3a**, **3b**, **4a**, **4b** and **5a**. These results suggest that the electron-withdrawing nitro group plays a crucial role in enhancing the observed activity. 

Compounds **3e**, **4c,** **4e** and **4f** showed a moderate activity (+ +) (MIC = 50 μg /mL) against *Staphylococcus*, while these compounds exhibited slight activity (+) (MIC = 75 μg/mL) against *Escherichia coli.* All other compounds were inactive towards the different strains of bacteria. The results are summarized in **Table 3**.

## 3. Experimental

### 3.1. General 

Melting points were taken in open capillary tubes using Electrothermal apparatus 9100 ( UK ) and are uncorrected. Microanalyses were performed at Faculty of Science, Cairo University, Cairo, Egypt, using a Elementary Vario el III C, H, N, S Analyzer (Germany). IR spectra were recorded using potassium bromide disks on a Perkin-Elmer 1650 spectrophotometer (Faculty of Science, Alexandria University, Alex, Egypt). ^1^H-NMR spectra were determined on a Varian EM-390 MHz spectrophotometer, using TMS as internal standard. 

### 3.2. General Procedure for Preparation of E-l-Cyclopropyl-3-(*p*-substituted-phenyl)-2-propenenones ***1a–c***

To a cold solution of sodium hydroxide (3 g) in aqueous ethanol (50 mL, 60%), cyclopropylmethyl ketone (10 mmol), was added dropwise (30 min), while rapidly stirring and the temperature kept below 20 °C, then the desired *p*-substituted benzaldehyde (10 mmol) was added dropwise (30 min). After five hours, the mixture was left overnight in refrigerator. The separated solid was filtered, washed with water and dried, then recrystallized from ethanol as colorless needles. The physical properties and all the spectral data were as reported in the literature [17].

### 3.3. General Procedure for Preparation of 1-Cyclopropyl-3-(*p*-substituted-phenyl)-2-propene-l-aroyl hydrazones ***2a–e***

A solution of chalcones **1a–c** (10 mmol) in ethanol (10 mL) was refluxed with the appropriate aroyl hydrazines (10 mmol) in glacial acetic acid (2 mL) for about six hours, then the reaction mixture was poured onto crushed ice and was kept overnight at room temperature, the separated solid was filtered off, washed successively with water and dried, then recrystallized from methanol. IR and NMR data: see **Table 1** and **Table 2**.

### 3.4. General Procedure for Preparation of 1-Aroyl-3-cyclopropyl-5-(*p*-substituted-phenyl)-pyrazoles ***3a–e***

A solution of the appropriate hydrazone **3a–e** (10 mmol) in 30% hydrochloric acid (15 mL) was refluxed for about two hours, the reaction mixture was concentrated, separated solid was filtered off, washed with water, dried and recrystallized from methanol. IR and NMR data: see **Table 1** and **Table 2**.

### 3.5. General Procedure for Preparation of 3-Acetyl-2-cyclopropyl-2-(*p*-substituted styryl)-5-(p-substituted phenyl)-1,3,4-oxadiazoles ***4a–e***

A mixture of the appropriate hydrazone **2a–e** (10 mmol) and acetic anhydride (15 mL) was heated under reflux for three hours. After the reaction mixture attained room temperature, it was poured into crushed ice and the oily product deposited was decanted from water and extracted with ether. The ether layer was washed with sodium bicarbonate, followed by water, dried over anhydrous sodium sulphate and evaporated to give the corresponding oxadiazoles **4a–e** as needles. IR and NMR data: see **Table 1** and **Table 2**.

### 3.6. General Procedure for Preparation of 3-Cyclopropyl-5-(*p*-substituted Phenyl)isoxazole ***5a–c***

A mixture of chalcone **1a–c** (20 mmol), hydroxylamine hydrochloride (20 mmol) and sodium acetate (20 mmol) in ethanol (25 mL) was refluxed for six hours. The mixture was concentrated by distilling out the solvent under reduced pressure and poured into ice-water. The precipitate obtained was filtered, washed and recrystallized from ethanol to give isoxazole **5** as needles. IR and NMR data: see **Table 1** and **Table 2**.

### 3.7. Determination of Antimicrobial Activity

All the synthesized heterocyclic compounds **3a–f**, **4a–f** and **5a–f** were tested against four different microorganisms: *Staphylococcus aureus*, *Escherichia coli*, *Pseudomonas aeruginosa* and *Candida albicans*. The *agar well-diffusion* method was applied for the determination of inhibition zone and minimum inhibitory concentration (MIC). Briefly, 0.75 mL of broth culture containing *ca.* 106 colon-forming units (CFU) per mL of the test strain was added to 75 mL of nutrient agar medium at 45 °C, mixed well, and then poured into a 15 cm sterile metallic Petri plate. The medium was allowed to solidify, and 8 mm wells were dug with a sterile metallic borer. Then, a DMSO solution of the test sample (1 mL) at 1 mg/mL was added to the respective wells. DMSO served as negative control, and the standard antimicrobial drugs rifampicin (5 μg/disc) and ampicillin (10 μg/disc) were used as positive controls. Triplicate plates of each microorganism strain were prepared and were incubated aerobically at 37 °C for 24 h. The activity was determined by measuring the diameter of zone showing complete inhibition (mm), thereby, the zones were precisely measured with the aid of a Vernier Caliper (precision 0.1 mm).The growth inhibition was calculated with reference to the positive control.

## 4. Conclusions

In summary, this work demonstrates a rapid, efficient method for synthesis of new heterocyclic compounds of pharmacological interest.

## Figures and Tables

**Table 1:** Physical and analytical data of compounds, **2a–f**, **3a–f**, **4a–f** and **5a–c**.

Compound	X	Y	Yield (%)	Mp (ºC)	Molecular Formula	Calculated %			Found %		
						C	H	N	C	H	N
**2a**	NO_2_	Me	72	181	C_20_ H_19_ N_3_ O_3_	68.77	5.44	12.03	68.71	5.39	12.09
**2b**	NO_2_	NO_2_	79	201	C_19_ H_16_ N_4_ O_5_	60.00	4.21	14.74	60.06	4.19	14.77
**2c**	H	H	66	166	C_19_ H_18_ N_2_ O	78.62	6.21	9.66	78.59	6.19	9.62
**2d**	H	Me	65	180	C_20_ H_20_ O N_2_	78.95	6.58	9.21	79.01	6.49	9.28
**2e**	OMe	Me	69	190	C_21_ H_22_ O_2_ N_2_	75.45	6.59	8.38	75.49	6.62	8.44	
**2f**	OMe	H	74	160	C_20_ H_20_ O_2_ N_2_	75.00	6.25	8.75	74.97	6.26	8.77
**3a**	NO_2_	Me	81	183	C_20_ H_17_ O_3_ N_3_	69.16	4.90	12.10	69.22	4.87	12.08
**3b**	NO_2_	NO_2_	92	197	C_19_ H_14_ O_5_ N_4_	60.32	3.70	14.81	60.33	3.69	14.77
**3c**	H	H	62	159	C_19_ H_16_ O N_2_	79.17	5.56	9.72	79.21	5.53	9.71
**3d**	H	Me	66	164	C_20_ H_18_ O N_2_	79.47	5.96	9.27	79.51	5.91	9.27
**3e**	OMe	Me	64	171	C_21_ H_20_ O_2_ N_2_	75.90	6.02	8.43	75.93	6.06	8.51
**3f**	OMe	H	59	180	C_20_ H_18_ O_2_ N_2_	75.47	5.66	8.81	75.52	5.60	8.86
**4a**	NO_2_	Me	83	201	C_22_ H_21_ O_4_ N_3_	67.52	5.37	10.74	67.52	5.43	10.80
**4b**	NO_2_	NO_2_	97	210	C_21_ H_18_ O_6_ N_4_	59.72	4.27	13.27	59.69	4.26	13.22
**4c**	H	H	59	177	C_21_ H_20_ O_2_ N_2_	57.90	6.02	8.43	57.84	5.99	8.44
**4d**	H	Me	58	179	C_22_ H_22_ O_2_ N_2_	76.30	6.36	8.09	76.28	6.31	8.12
**4e**	OMe	Me	67	189	C_23_ H_24_ O_3_ N_2_	73.40	6.38	7.45	73.44	6.39	7.49
**4f**	OMe	H	80	162	C_22_ H_22_ O_3_ N_2_	72.93	6.08	7.73	72.91	6.01	7.76
**5a**	NO_2_	―	54	165	C_12_ H_10_ O_3_ N_2_	62.61	4.35	12.17	62.66	4.29	12.21
**5b**	H	―	49	159	C_12_ H_11_ O N	77.84	5.95	7.57	77.90	5.99	7.62
**5c**	OMe	―	74	161	C_13_ H_13_ O_2_ N	72.56	6.05	6.51	72.60	6.01	6.48

**Table 2:** IR and ^1^H-NMR spectral data of compounds **2a–f**, **3a–f**, **4a–f** and **5a–c**.

Compound	IR cm^−1^ (KBr)				^1^H NMR (δ / ppm)^a^						
	C=C	C=N	C=O	NH	Ar–H’s and = C–C*H*=CH (m)	=C–CH=C*H* (d), *J* =12 Hz	Pyrazole-C_4_–H (s) or isoxazole–C_4_–H (s)	NH (s)	Cyclopropyl ring H’s		Ar–CH_3_ (s) Ar–OCH_3_ and CH_3_CO–
									CH (m)	2 (CH_2_) (m)	
**2a**	1592	1629	1678	3420	7.36–7.91	6.72	―	10.66	1.91–2.55	0.75–1.37	2.21
**2b**	1617	1647	1698	3390	7.23–8.45	6.93	―	10.45	1.80–2.64	0.71–1.40	―
**2c**	1582	1616	1664	3330	7.19–7.63	6.60	―	9.97	1.82–2.49	0.78–1.29	―
**2d**	1587	1636	1669	3336	7.29–7.82	6.71	―	10.82	1.71–2.36	0.70–1.40	2.10
**2e**	1601	1628	1687	3411	7.20–7.71	6.75	―	10.73	1.63–2.40	0.77–1.46	2.19, 3.49
**2f**	1617	1644	1681	3332	7.17–7.49	6.95	―	9.99	1.56–2.59	0.78–1.16	3.42
**3a**	1519	1629	1670	―	7.26–7.92 ^b^	―	6.85	―	1.90–2.48	0.69–1.21	2.13
**3b**	1586	1633	1684	―	7.24–8.33 ^b^	―	7.12	―	1.76–2.66	0.80–1.36	―
**3c**	1556	1641	1660	―	7.22–7.71 ^b^	―	6.78	―	1.73–2.40	0.71–1.28	―
**3d**	1590	1644	1678	―	7.24–7.77 ^b^	―	6.64	―	1.75–2.41	0.77–1.33	2.11
**3e**	1571	1623	1686	―	7.21–7.68 ^b^	―	6.69	―	1.66–2.39	0.71–1.33	2.10,3.44
**3f**	1596	1633	1664	―	7.11–7.62 ^b^	―	6.74	―	1.49–2.55	0.77–1.19	3.39
**4a**	1587	1645	1669	―	7.18–7.79 ^b^	―	―	―	1.92–2.41	0.67–1.32	2.01, 2.16
**4b**	1610	1646	1667	―	7.22–8.22 ^b^	―	―	―	1.70–2.66	0.71–1.40	2.13
**4c**	1602	1626	1669	―	7.16–7.75 ^b^	―	―	―	1.68–2.39	0.75–1.26	2.11
**4d**	1594	1630	1670	―	7.33–7.76 ^b^	―	―	―	1.73–2.44	0.71–1.38	1.99, 2.13
**4e**	1600	1633	1677	―	7.26–7.72 ^b^	―	―	―	1.60–2.47	0.71–1.26	2.13,3.32,2.15
**4f**	1615	1646	1671	―	7.25–7.51 ^b^	―	―	―	1.47–2.61	0.76–1.33	3.34,2.10
**5a**	1580	1627	―	―	7.19–7.99 ^b^	―	5.69	―	1.82–2.51	0.71–1.36	―
**5b**	1571	1629	―	―	7.22–7.62 ^b^	―	5.42	―	1.43–2.45	0.69–1.27	―
**5c**	1569	1633	―	―	7.26–7.70 ^b^	―	5.46	―	1.57–2.41	0.67–1.29	3.30

^a^ Solution in DMSO-d_6_; ^b^ The chemical shift only indicates Ar–H’s.

**Table 3:** Antibacterial activities of newly synthesized compounds 3–5.

Compound	X	Y	*Staphylococcus aureus*	*Escherichia coli*
**3a**	NO_2_	Me	+ + +	+ + +
**3b**	NO_2_	NO_2_	+ + +	+ + +
**4a**	NO_2_	Me	+ + +	+ + +
**4b**	NO_2_	NO_2_	+ + +	+ + +
**5a**	NO_2_	―	+ + +	+ + +
**3e**	OMe	Me	+ +	+
**4c**	H	H	+ +	+
**4e**	OMe	Me	+ +	+
**4f** **3c** **3d** **3f** **4d** **5b** **5c**	OMe H H OMe H H OMe	H H Me H Me ― ―	+ + ― ― ― ― ― ―	+ ― ― ― ― ― ―

+ + + for maximum activity, MIC = 25 μg/mL; + + for moderate activity, MIC = 50 μg/mL; +for slight activity, MIC = 75 μg/mL and – for inactive.
